# Exploring the Emerging Domain of Research on Video Game Live Streaming in Web of Science: State of the Art, Changes and Trends

**DOI:** 10.3390/ijerph18062917

**Published:** 2021-03-12

**Authors:** Luis Javier Cabeza-Ramírez, Fernando J. Fuentes-García, Guzmán A. Muñoz-Fernandez

**Affiliations:** Faculty of Law, Business and Economic Sciences, University of Córdoba, Puerta Nueva s/n, 14071 Córdoba, Spain; fernando.fuentes@uco.es (F.J.F.-G.); guzman.munoz@uco.es (G.A.M.-F.)

**Keywords:** live video streaming, video game live streaming, literature review, bibliometric, communication process

## Abstract

In recent years, interest in video game live streaming services has increased as a new communication instrument, social network, source of leisure, and entertainment platform for millions of users. The rise in this type of service has been accompanied by an increase in research on these platforms. As an emerging domain of research focused on this novel phenomenon takes shape, it is necessary to delve into its nature and antecedents. The main objective of this research is to provide a comprehensive reference that allows future analyses to be addressed with greater rigor and theoretical depth. In this work, we developed a meta-review of the literature supported by a bibliometric performance and network analysis (BPNA). We used the PRISMA (Preferred Reporting Items for Systematic Reviews and Meta-Analysis) protocol to obtain a representative sample of 111 published documents since 2012 and indexed in the Web of Science. Additionally, we exposed the main research topics developed to date, which allowed us to detect future research challenges and trends. The findings revealed four specializations or subdomains: studies focused on the transmitter or streamer; the receiver or the audience; the channel or platform; and the transmission process. These four specializations add to the accumulated knowledge through the development of six core themes that emerge: motivations, behaviors, monetization of activities, quality of experience, use of social networks and media, and gender issues.

## 1. Introduction

Video games go beyond being a simple hobby linked to young people and adolescents; instead, they have become a new form of adult leisure activity commonplace in contemporary society [1]. They comprise an independent industry within the cultural sector, generating ever-greater revenues and yielding a sophisticated product at the philosophical, sociological, aesthetic, cultural, or narrative level [2,3]. Their remarkable evolution over time has seen them go from being simple games primarily played alone to becoming products that allow millions of users to socialize and share experiences online [4,5]. Video games are currently undergoing a genuine transformation thanks to two relatively recent and interconnected phenomena: the professionalization of gaming through e-sports [6], and the viewing of live content through live streaming services [7,8,9]. Recent years have witnessed the expansion of the video game live streaming industry and the emergence of new platforms that offer this type of service, most notably Twitch [10].

The first academic papers on video game live streaming were relatively new. Li et al. [11] and Hamilton et al. [12] pointed to the analysis carried out by Kaytoue et al. [13] as an early contribution to the literature. In turn, that study highlighted others that have tied the phenomenon to the rise of social television [14], or the success of platforms such as YouTube [15]. These pioneering studies offer an understanding of this new form of leisure activity. However, perhaps due to the relative recency of the related academic literature or the markedly interdisciplinary nature of the phenomenon, there is currently a degree of confusion surrounding the different terms and definitions used to refer to the same activity. For example, Zheng et al. [16] use the term crowdsourced live game video streaming (CLGVS) to refer to platforms that allow a mass audience to receive or stream content from other sources, combining crowdsourcing technology with live video game streaming. Gros et al. [17] and Zimmer et al. [18] emphasized the social functions of the platforms using the term *social live streaming services* (SLSSs) to refer to a new type of social network with specific features such as synchronization, real-time streaming, viewer–streamer interaction, and a rewards system. For their part, Ma et al. [19] underlined the interactivity of the service using the term live broadcast with community interactions to highlight the combination of high-quality video game graphics, real-life activities captured by webcam and open chat channels for viewers to interact with each other or with the streamer. Conversely, Barman et al. [20] referred to such services as passive gaming video streaming, thereby distinguishing them from interactive cloud applications used by gamers to play online, and underscoring the apparently passive role of the viewer when watching others play. More recently, the first review to specifically focus on the behavior of users of video game live streaming services defined them as “*a form of media integrating the public, communities, interaction, and passivity, while bridging the gap between online games and traditional video media (such as TV)*”, and regarding the content, stated that “*video game live streaming is defined as a network broadcast with ‘online games’ as the specific content*” [11]. 

Besides the definition or terminology used, there are certain common features that have emerged in most of the related publications to date. One is the importance of the process of communication for understanding the phenomenon of video game live streaming; another is the central role played by the activity of video games, whether active participation—development and streaming—or passive—watching without taking part [20]. The present study aims to explore in more depth the emerging domain of research on video game live streaming. To do so, we take as our starting point the main elements represented in the mathematical theory of communication [21]. We propose a theoretical framework, which we use to classify the existing literature according to its characteristic components: transmitter or streamer; channel or streaming platform; receiver or audience; and a fourth category made up of studies that primarily address the streaming itself including the code used and the messages conveyed. Therefore, the main aim of this research is to provide a comprehensive view of the research domain, allowing future analyses to be carried out with greater rigor and theoretical depth. To that end, we conducted a systematic literature review [22] supported by bibliometric analysis. In addition, we present the main research topics studied, which allows us to identify future research challenges and trends. The few existing reviews thus far have only addressed very specific aspects such as user behavior [11] or the development of tools to optimize the research [23]; however, we have not found any studies to date that have explored the existence of a new domain of research and its key constituent topics—a gap this study aims to fill. To do so, we applied the PRISMA protocol [24] and carried out a Bibliometric Performance and Network Analysis (BPNA) to evaluate the impact of the phenomenon. We then used the VOSviewer [25] software to visualize the main thematic networks identified. 

## 2. Method

Three complementary methodological approaches were combined in order to achieve the proposed research objectives. First, we applied the PRISMA protocol [22,24] designed for systematic literature reviews and meta-analyses. Based on an exhaustive review of the literature, the research domain was defined and a common theoretical framework was identified. Second, bibliometric performance analysis (BPNA) was carried out in order to assess the impact of the scientific output of different contributors [26]. Finally, science mapping through a keyword co-occurrence analysis revealed the thematic and intellectual structure of video game live streaming research, [27,28]. According to Onwuegbuzie et al. [29], the blend of qualitative data, in this case derived from the review and culminating in scientific mapping in combination with quantitative bibliometric data, contributes specifically to the explanation of patterns within a given field, discipline, or body of knowledge (i.e., the quantitative stages help to develop the patterns previously detected in the qualitative phase). The main features of the method applied are detailed below. 

### 2.1. Systematic Literature Review

The PRISMA protocol [24] is a proven methodological procedure that helps ensure research is transparent and replicable [30]. It has previously been used in the field of video games [31] as well as in research specifically focused on live streaming [11,23]. It consists of four key stages: 

Identification: The present study used a single source of information, Web of Science (WoS). WoS is a data source owned by Clarivate Analytics that includes several databases; in our work, we used the Core Collection and the citation indexes: Science Citation Index Expanded (SCI-EXPANDED) 1900–present; Social Sciences Citation Index (SSCI) 1956–present; Arts & Humanities Citation Index (A&HCI) 1975–present; Conference Proceedings Citation Index-Science (CPCI-S) 1990–present; Conference Proceedings Citation Index-Social Science & Humanities (CPCI-SSH) 1990–present; Book Citation Index-Science (BKCI-S) 2005–present; Book Citation Index-Social Sciences & Humanities (BKCI-SSH) 2005–present; Emerging Sources Citation Index (ESCI) 2005–present. Although this represents a limitation in terms of the coverage of documents in the review, it ensures a single format for citation, usage, and classification in areas of research. WoS offers an adequate volume of documents, proven scientific quality, and data cleaning to correct errors [32]. The selection of search terms was based on previous research and included different combinations: [11,12,23]: “live streaming”; “live-streaming”; “game streaming”; “game-streaming”; “social live streaming”; “internet broadcast”; “network broadcast”; “webcast”; “twitch”; “twitch.tv”; “youtube live”. In terms of time coverage, following Li, Wang, and Liu [11], the decision was made to include all published documents written in English from 2010, two years prior to the appearance of Twitch. No types of document were excluded, which made it possible to include papers from conferences and other sources such as books, or chapters thereof, which may be of interest in the analysis of emerging research domains.

Screening: The resulting searches were refined and filtered using the term “video game” and its plural. The titles, keywords, and abstracts of the documents were reviewed. At this initial stage, documents that indicated that the research was about live streaming were kept and those that clearly did not address the specific topic, gaming or video games, were removed. Records that could not be discarded without a full-text reading were retained. The references contained in the main studies were tracked and the results cross-checked against previous reviews [11,23]. This made it possible to identify new items that had not been detected with the initial search sequence.

Eligibility: The criteria applied were, first, that the main topic of the document directly addressed video game live streaming, even if it was as part of a broader study; second, that the document was indexed in the WoS and had undergone some type of peer review process as an article or text from a conference, or was editorial material indexed in the WoS; and third, that it was possible to retrieve the full-text document. All references resulting from the application of these criteria were downloaded. 

Included: The full-text documents were read excluding those linked to live streaming that did not specifically deal with games or video games, those whose main topic was not clear, those that did not present any research data, or those whose methodology could not be deduced specifically from the text. 

**Figure 1:**
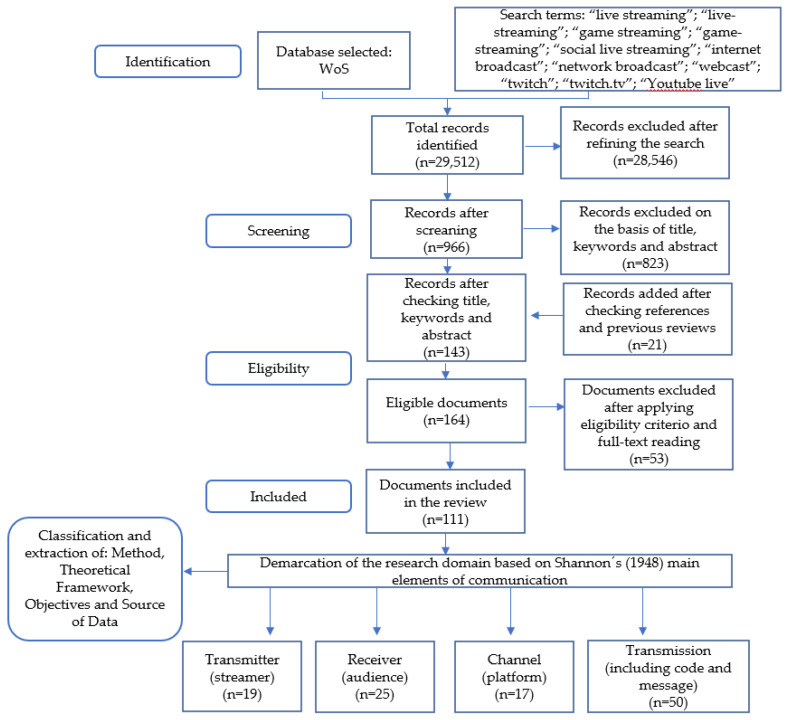
Flow diagram adapted from the Preferred Reporting Items for Systematic Reviews and Meta-Analysis (PRISMA) protocol.

The search for and reading of references yielded 111 documents. The process started in September and was continuously updated to include new records indexed in the WoS until 1 December 2020. From the full-text reading, the different definitions provided for the phenomenon of video game live streaming were extracted [11,17,18,19,33]. On the basis of those definitions, and after verifying how the communication process was addressed directly or indirectly in all the identified documents, they were classified in relation to the main elements of the mathematical theory of communication proposed by Shannon [21]. The complete process is detailed in Figure 1. The methodological approach applied, theoretical framework, objectives, and data source were extracted from each of the documents. 

Documents were classified according to the main focus of the research. In line with previous studies [11,23] and based on the basic elements of the communication process [21], it was determined whether the document was essentially focused on the Transmitter/Streamer (S), Receiver (A), Platform (P), or Transmission including the codes and the message (T). In cases where a dual focus was observed, for example, streamer and audience, the document was classified according to the predominant focus and this was specifically indicated. The methodology used was coded to distinguish between quantitative (Qn), qualitative (Ql), and mixed (M) methods [34], and technical (Tc) [35] studies (technical Human–Computer Interaction (HCI) research includes studies directly contributing to meeting some human need as well as studies indirectly contributing by enabling other technical work through things like toolkits), and a brief explanatory note was provided. The theoretical framework was then indicated, differentiating between documents that rely on established theories and those that do not use defined theories, but rather are based on previous research. Finally, the abstract was extracted with the objectives of the document on which to base the subsequent thematic classification as well as the source of the data used in each paper.

### 2.2. Bibliometric Performance Analysis

The study was based on a number of indicators: direct citation, h-index of the categories considered (streamer, audience, platform, and transmission) as well as usage counts since 2013 and in the last 180 days. Citation is used as a measure of the relative influence of publications; when a document is highly cited, it can—with the appropriate caveats [36]—be considered important for the research domain [28]. The Hirsch Index or h-index [37] was originally designed to measure researchers’ scientific performance. In this study, it was used to evaluate sub-areas of knowledge [38,39]. Research subdomains have an h-index of h if the h of their Np (total number of publications) have at least h citations each, and the other publications (Np-h) have at most h citations each [40]. As for usage counts, they are considered as an early measure of the attention a certain document receives, either through clicks or downloads. In WoS, they occur when a user tries to download the full text or saves the record in a bibliographic management tool; that is, they in some way reflect potential future citations, or at least capture the initial impact of the text [41]. The different analyses were performed using the Analyze Results tool available in WoS. The bibliometric approach yields lists or tables with the main documents, indexing categories, type of document, country of origin, and journals within the domain under study. The h-index, citations, and usage counts were up to date as of 28 December 2020. 

### 2.3. Science Mapping

Science mapping produces a spatial representation of the relationships among a group of documents, authors, journals, disciplines, or research domains. The analysis of scientific networks has previously been used in different fields to visualize these types of relationships [28,42]. In line with the research objective, the network analysis focused on the content of the documents through keyword co-occurrence. As pointed out by Chen et al. [43] and Zupic and Cater [28], this is a particularly appropriate method for identifying early on the intellectual structure of a given research domain. Furthermore, Choi et al. [44] stated that the keywords and titles of the documents are crucial for identifying significant topics. The network of relations between terms is visualized using the open source software VOSviewer [25]. The centrality of a node (word) indicates its relative position in the network: the software calculates the centrality and strength of the different nodes and links, thereby revealing the thematic structure of the domain. Nodes depict the appearance of keywords; the bigger the node, the more important the item. Links between nodes represent the number of times keywords appear together. The strength of the link is illustrated by its thickness. As this is an emerging domain with a sample of only 111 documents, a minimum of n = 2 occurrences was set for the domain as a whole and n = 1 for the four subdomains (streamer, audience, platform, and transmission), thus ensuring a complete visualization of all the identified thematic networks. A thesaurus file was used for data cleaning and to group synonymous terms or the singular and plural of certain words: for example, *electronic sport*, *esports,* and *e-sport game* were all introduced under the word *e-sport*. The other parameters used in VOSviewer are detailed in Table 1. 

## 3. Results

This study adopted a broad definition of research domain: *“thought or discourse communities, which are parts of society’s division of labor”* [45]. As Hjorland and Hartel [46] argue, research domains are composed of ontological theories and concepts about the objects of human activity; epistemological theories and concepts about knowledge and the ways to obtain it, or methodological principles; and sociological aspects relating to the groups of people concerned with said objects.

### 3.1. The Domain of Research on Video Game Live Streaming

A research domain arises from one or more academic fields that expand according to interests, themes, values, or conflicting points of view or values, with the academics involved moving toward specific sub-communities [47,48]. In order to demarcate the research domain on video game live streaming, we referred to previous studies that sought to explore the boundaries of specific thematic areas [49]. According to Shirmohammadi, Mehdiabadi, Beigi, and McLean [47], an initial approach entails asking four basic questions: what, how, who, and where? The answer to those questions contributes to a better understanding of the phenomenon and its main players. The literature review helps to answer the “what” and the “how”; the subsequent BPNA tells us the “who” and the “where”. 

With regard to the essence of the phenomenon—the “what”—the application of the PRISMA protocol [24] yielded 111 documents, which made it possible to outline specific features of the research domain. The accumulated research included studies of social networks [18], video games [8,50], and media [9,51]. A feature that all the documents have in common is the communication process arising from the different definitions and meanings developed so far on the concept of video game live streaming [11,16,17,19,52]. Accordingly, the accumulated research can be represented through the classic diagram produced by Shannon [21]. That is, a transmitter or streamer transmits a multitude of messages (text, image, video, sound) essentially related to the active practice of video games; thus, the documents that fundamentally focus on the figure of the transmitter or streamer can be grouped together. Messages are encoded and decoded through a platform, acting as a transmission channel, which mediates the relationship between the transmitter and the receiver as well as the relationships among receivers; this provides the basis for grouping together the articles that primarily deal with the transmission platform itself. When messages (source of information) reach their destination, a receiver or group of receivers (audience) transmit new messages (chat, gifts, icons, subscriptions, follows, etc.) to the receiver, to each other, or they remain passive, simply receiving content without reacting to it; thus, there is another group of documents focused mainly on the audience. The entire process is characterized as being interactive, practically instantaneous, and with immediate feedback, but not free from noise (overload of comments in chats, problems with the quality of video or audio, etc.); therefore, the last group that emerges primarily addresses the code, the messages, or the transmission process itself.

**Figure 2:**
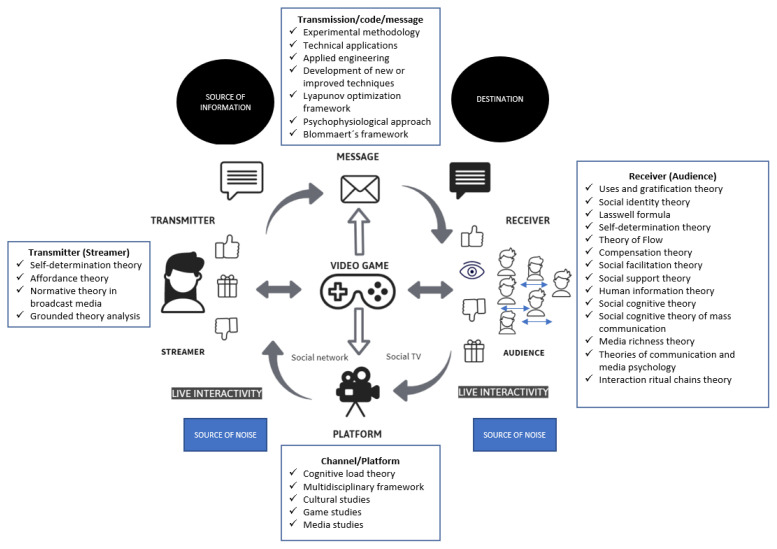
Research domain and main theories applied.

As for the “how”, the domain of the accumulated research on video game live streaming is constructed through the application of various theoretical frameworks. Although many of the papers did not provide information on the theories they reference and are broadly based on previous studies, it is possible to identify certain established frameworks that are repeatedly used. Within the overall domain, the four above-mentioned subdomains or areas of specialization can be distinguished. The group of documents or subdomain focused primarily on the streamer employed well-established theories such as self-determination theory [53,54], affordance theory [10], normative theory of broadcast media [55], or grounded theory methodological analyses [6,56,57,58]. The second subdomain, focused on the receiver or the audience, was clearly underpinned by widely-tested theories: uses and gratifications theory [7,11,17,18,59,60,61,62,63,64], social identity theory [65], Lasswell formula [18], self-determination theory [18,62,66], theory of flow [18], compensation theory [67], social facilitation theory [62], social support theory [68], human information theory model [69], social identity theory [69], media richness theory [63], social cognitive theory of mass communication [70], and interaction ritual chains theory [33]. The third group, focused on the channel, relied less on well-established frameworks, mostly drawing on previous studies. However, we observed the application of multidisciplinary frameworks incorporating cultural studies, game studies, and media studies [52], cognitive load theory [71], and the development of the ‘platform’ concept or the ‘actor–network theory’ [72]. Finally, the fourth group of papers, which mainly addressed the transmission itself, largely consisted of technical documents [35] that sought to improve transmission or provide solutions relating to the video, code, or the message. These studies mostly come from two areas of research: computer science and engineering. Their focus is more on providing solutions to problems, as such, they particularly rely on experiments [73,74,75], the development of applications, and applied engineering [76,77,78,79]. Therefore, they do not need a strong grounding in well-established theories, although some do occasionally appear; for example, the Lyapunov optimization theory [16], the psychophysiological approach [80], Blommaert’s framework of critical discourse analysis, and Computer-mediated discourse analysis (CMDA) [81]. Figure 2 depicts the entire domain and the most commonly-used frameworks for improving the knowledge base.

Regarding the discipline and the methodology, Table 2 presents for each of the identified research subdomains the main approaches applied and the research areas in which the WoS indexes these documents. In the subdomain focused on the streamer, it can be seen that qualitative methods predominate, especially exploratory studies, interviews, or ethnographic methods [6,10,56,57,58,82,83,84], with samples ranging from 20 [6] to 100 interviews [57,58,82] and observational data from 20 to 70 hours of live streaming [56,85]. This may be partly due to the difficulty of obtaining a broad database with enough streamers to carry out the type of quantitative analysis that represents the traditional methodological approach in the predominant research area (communication). Moreover, qualitative analysis seems particularly suitable for gaining a deeper understanding of complex processes such as the creation of live content or the professionalization of these activities [10,58]. Research taking a quantitative approach is less common, although it includes studies such as those of Zhao, Chen, Cheng, and Wang [53] and Torhonen, Sjoblom, Hassan, and Hamari [54], who applied structural equation modeling and analyzed samples of up to 377 content developers. Conversely, in the subdomain focused on the audience, quantitative research prevails, with many studies using structural equation models [7,59,60,63,64,65,66,70,86]. Sample sizes ranged from 412 users [65] to more than 1000 [60,64], even reaching 2227 Twitch users [61] in a study conducting multiple and ordinal linear regression analyses. For this second group of studies, it was easier to achieve broad samples of the audience (potential or real) through social networks and user forums. A substantial number of these papers have their roots in the discipline of psychology, which has a long tradition of developing and using models to explain behavior [59,60,65]. The subdomains focused on the platform and the transmission are characterized by the use of more heterogeneous methods, although the former stands out for the inclusion of descriptive statistics and techniques for constructing graphs [87,88,89,90,91,92], and the latter for compiling information through application programming interfaces (APIs), both for Twitch [73,93,94,95,96,97] and other platforms such as Douyu [98]. The collection of information through public APIs is spreading to other subdomains (Streamer [99] Riot Games API; Receiver [100] Twitch API; and Platform [87,89,91] Twitch API), opening up future research opportunities if simultaneous information about the streamer, receiver, and platform can be connected.

**Table 2:** Main methodology and research area.

	Streamer Subdomain	Audience Subdomain	Platform Subdomain	Transmission Subdomain
Qn	[53,54,99,101,102]	[7,17,33,59,60,61,63,64,65,66,67,70,86,100,103,104,105,106,107]	[71,87,88,89,90,92,108,109]	[97,110,111,112,113,114]
Ql	[6,10,55,56,57,58,82,83,84,85,115,116]	[18,62]	[8,9,117,118,119]	[81,120,121,122,123,124,125,126]
M		[11,68,69,127]	[23,52,72]	
Tc	[128,129]		[91]	[16,19,20,73,74,75,76,77,78,79,80,93,94,95,96,98,130,131,132,133,134,135,136,137,138,139,140,141,142,143,144,145,146,147,148,149]
Research area (nº doc ≥ 5)	Communication 9; Computer Science 7;	Computer Science 10; Psychology 7	Computer Science 9; Telecommunications 5	Computer Science 37; Engineering 18; Telecommunications 13

### 3.2. Bibliometric Performance Analysis of the Research Domain

The “who” and the “where” were answered by examining the main players through the bibliometric analysis of the 111 documents in the review. Although the period of analysis started in 2010, the first studies did not appear until 2012. These were technical papers focused on developing new systems that allow video to be shared with players online [130], lab experiments with traditional games converted into video games to show how watching someone play live provides a very similar experience to face-to-face playing [74], or simulations to try to reduce bandwidth overhead and improve the broadcast [131]. These are seminal papers, published at conferences and belonging to the fields of computer science, engineering, and telecommunications. Although these are the studies that have been available for the longest time, their impact in terms of citations or downloads has been limited, probably due to the fact that they are preliminary studies presented at conferences. 

**Figure 3:**
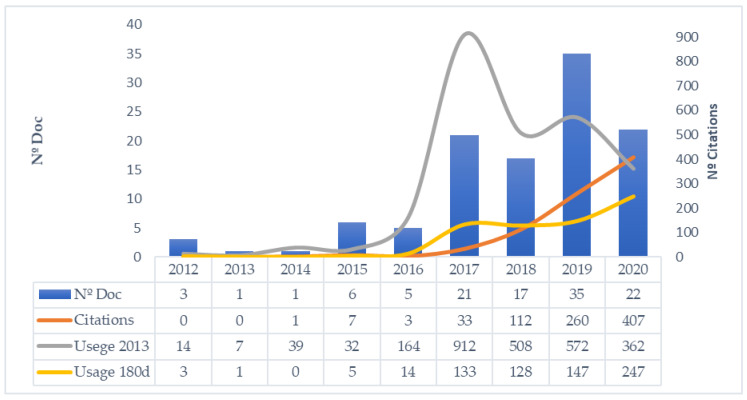
Number of documents and impact in terms of citation and usage.

In Figure 3, it can be seen how the number of documents related to video game live streaming started to increase in 2015, although they were still mostly from conferences [87,88,103,110,132,133]. Among them were the first analyses of specific platforms such as Twitch [87,110,132] or YouNow [88]. The predominant research area is still computer science. Some of these conference papers [88] managed to attract attention from other specialized fields such as communication and social sciences, thus increasing citations and boosting interest in the domain.

From 2017, the number of publications grew exponentially before levelling off at an average of around 20 documents per year. The research generated has attracted attention from other areas of knowledge such as psychology [59,60,65,71], sociology [120], or linguistics [121]. The study of the phenomenon of live streaming becomes multidisciplinary and there is an increase in both the citations of all these new publications and accumulated downloads. Indeed, from this moment on, we observe the emergence of identifiable lines or subdomains of research on the streamer [6,53,54], the audience [86,106,107], the platform [9,71,117], and the streaming process [16,19,95]. A ranking of the top three publications in each of the subdomains is included in Table A1, Appendix A. In turn, there is greater heterogeneity in terms of themes, for example, studies emerge on professional streaming on Twitch [55], information behavior, and the users’ perceptions of co-presence on Twitch [69], the gambling engagement mechanisms in live streaming services [118], or sexual content policies applied on the Twitch platform [126]. Table 3 presents a summary of the impact measures considered. 

**Table 3:** Main bibliometric indicators for the analyzed sample.

Streamer Subdomain	Nº Doc.	h-Index	Average Citations per Item	Sum of Times Cited	Usage Count Since 2013	Usage Count Last 180 Days
[6,10,53,54,55,56,57,58,82,83,84,85,99,101,102,115,116,128,129]	19	7	6	114	514	141
Audience Subdomain						
[7,11,17,18,33,59,60,61,62,63,64,65,66,67,68,69,70,86,100,103,104,105,106,107,127]	25	8	16.44	411	1217	317
Platform Subdomain						
[8,9,23,52,71,72,87,88,89,90,91,92,108,109,117,118,119]	17	7	5.88	100	399	102
Transmission Subdomain						
[16,19,20,73,74,75,76,77,78,79,80,81,93,94,95,96,97,98,110,111,112,113,114,120,121,122,123,124,125,126,130,131,132,133,134,135,136,137,138,139,140,141,142,143,144,145,146,147,148,149]	50	7	3.96	198	480	118
Video Game Live Streaming Services Domain	111	15	7.41	823	2610	678

To put these values into context, it should be noted that although the audience subdomain registered the highest values, it was also the specialization that accounted for the greatest number of documents belonging to disciplines aligned with the study of behavior [7,59,61]. This type of research was found to be the most cited, displaying a remarkable evolution with respect to the time of exposure to the citation. At the same time, these were the publications that registered the most downloads. These focused on shedding light on the underlying reasons of why a growing audience used this type of service and watched others playing. It should be pointed out that these indicators have not been normalized; in other words, they do not account for the differences in citation and publication practices between scientific fields [27]. Overall, we observed an h-index of around 15 and practically identical values in the different lines of specialization. This could represent a potential boost for the future influence of the domain. As it starts to grow, new areas of knowledge become involved in the domain and the results are transferred between those areas of knowledge. 

Moreover, the sample of publications was composed of a large number of proceedings papers, a clear indication of its relative recency. We should expect to see a significant proportion of these preliminary articles evolving and being refined [20,130,139,140]. Table 4 presents a summary of the most relevant information about the general profile of the domain. There were signs of specialization with authors such as Juho Hamari, Mark R. Johnson, or Max Sjoblom, who each had more than six papers to their name.

**Table 4:** General document profiling of research on video game live streaming services.

Category	Items
WoS Categories (no. publications ≥)	Computer Science Theory methods 36; Computer Science Information Systems 27; Engineering Electrical Electronic 22; Telecommunications 20; Computer Science Software Engineering 19; Communication 17; Computer Science Cybernetics 10; Computer Science interdisciplinary applications 10; Computer Science Artificial Intelligence 9; Psychology Multidisciplinary 9; Psychology Experimental 9; Computer Science Hardware Architecture 6; Sociology 6; Business 3; Environmental Sciences 3; Imaging Science Photographic Technology 3; Multidisciplinary Sciences 3; Public environmental Occupational health 3; Cultural Studies 2; Film Radio Television 2; Information Science Library Science 2; Language Linguistics 2; Linguistics 2; Behavioral Sciences 1; Education Educational Research 1
Document Types	Article 62; Proceedings paper 47; Early Access 3; Book Chapter 1; Editorial material 1; Review 1
Most Prolific Authors (no. publications ≥ 4)	Hamari, Juho. Tampere University 6; Johnson, Mark R. Alberta University 6; Sjoblom, Max. Tampere University 6; Liu Jiangchuam. Simo Fraser University 5; Barman, Nabajeet. Kingston University 4; Martini María G. Kingston University 4; Woodcok, Jamie. Open University 4; Zhang, Cong. University of Science & Technology of China 4
Most Productive Sources Titles (no. publications ≥ 3)	Computers in Human Behavior 8; Lecture Notes in Computer Science (conferences) 8; IEEE Access 3; IEEE Transactions on Circuits and Systems for Video Technology 3: Information Communication Society 3; Multimedia Tools and Applications 3; New Media and Society 3
Most Productive Institutions (no. publications ≥ 5)	Simon Fraser University 7; University of California System 7; Tampere University 6; Aalto University 5: University of Alberta 5
Most Productive Countries (no. publications ≥ 4)	USA 41; China 18; Canada 15; England 12; Germany 12; Taiwan 8; Finland 7; Australia 4: Qatar 4; South Korea 4
Core References (no. of received citations ≥ 15; citations and subdomain)	[7] 128-A; [59] 79-A; [61] 52-A; [65] 43-A; [60] 31-A; [53] 25-S; [112] 24-T; [52] 22-P; [17] 22-A; [101] 18-S; [71] 16-P; [121] 16-T; [16] 16-T; [120] 16-T;

The WoS categories also pointed to a gradual increase in the number of research areas involved. It should be borne in mind that the same document can be classified in more than one category depending on the indexing of the journal or the conference in which it appears. Interestingly, despite the disruption to the video game industry [8] caused by live streaming services, only three documents were catalogued under business [7,54,105]. This represents an opportunity for future research whereby new approaches from the fields of management or economics can be applied to complement or corroborate previous findings [55,58,82]. The same is true of the other as yet underexplored categories such as the application of linguistics to text analyses of chats, cultural studies on the new communities being created, or environmental studies to assess the impact of this new industry. The present review did not find any papers specifically focused on the sound or transmission of audio. Thus far, it seems that the phenomenon of video game live streaming has had the most notable impact in specialized journals in the field of behavioral studies and psychology; indeed, the journal Computers in Human Behavior has already published eight related papers. This is followed at some distance by technical journals such as IEEE Access, and those linked to communication and society such as Information Communication Society and New Media and Society. On the other hand, if we observe the number of documents in the sample published in journals included in Journal Citation Report (JCR), almost 64% reached journals positioned among the first quartiles; the remainder corresponded mainly to papers submitted to congresses, which are likely to be improved and published in JCR categories, as previously mentioned. Table A2 includes the list of papers, the journal of publication, and the quartile.

### 3.3. Thematic Structure of the Research Domain

After using the VOSviewer thesaurus file to clean the keywords, we were left with a total of 390, of which only 85 met the established criterion of a minimum of two occurrences. The software calculated the total strength of the co-occurrence links between terms, and selected those with the highest total link strength. The full list, along with the thematic clusters, links, and occurrences, is presented in Table 5. For each of the resulting thematic clusters, the most representative keywords were chosen to designate each group. The clusters were composed of some generic terms directly related to the domain itself such as video game, crowdsourced live video, or live platform; however, in each case, an identifiable thematic focus was observed. The thematic cluster analysis was verified by examining the objectives and the results of each of the documents included in the sample.

**Figure 4:**
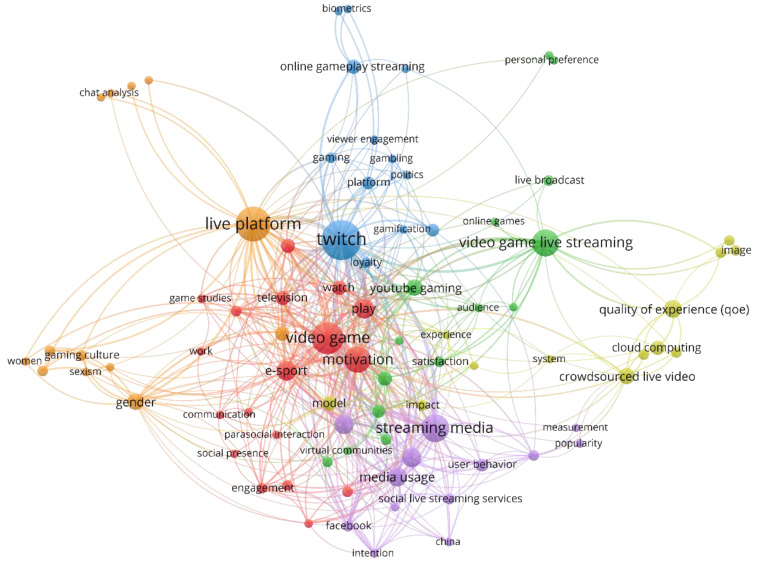
Map of thematic clusters identified in the research domain on video game live streaming.

Figure 4 shows the 85 terms used for the visual representation of the analysis as well as the six thematic clusters detected with the network visualization tool VOSviewer. The relationships between clusters and the nodes belonging to each of the clusters are depicted in the distances between the different elements: the shorter the distance, the greater the interdependence between the detected clusters [25]. 

The thematic clusters related to motivation (C1), behavior (C2), monetization of activities (C3), and media usage (C5) predominated in the areas of specialization focused on the streamer and the audience. Furthermore, they were the ones that had had the greatest impact in terms of citations and downloads. Conversely, the thematic clusters focused on the quality of the experience (C4), and gender and content (C6) had a stronger presence in the subdomain focused on transmission. While their impact has been less marked, it is expected to increase as connections develop between the accumulated knowledge in the different specializations. Finally, documents covering the channel or the transmission platform appeared across the six thematic clusters identified, and achieved an average impact relative to the other specializations detected. 

Subsequently, the main topics on video game live streaming obtained in each cluster are detailed. The first cluster contains topics relating to the motivations for watching and streaming, and to the consumption of e-sports, work, and the digital economy. This cluster includes studies focused on the different motivations that drive the audience [7,17,64] and content developers or streamers [53,54,85]. It also covers the characteristics, motivations for, and challenges of live-stream programming [85], new forms of paid work [6,10,55,56,57,58,83], user motivation [7,17], and identification between audience groups and streamers [65]. The relationships between the genre played, content streamed and viewer gratification [59], the motivations for spending money [104], and the relationships between the size of the channel and user motivation [61] are also explored. In addition, this cluster addresses the possible differences in said motivations according to the gender of the viewer or the streamer [105]. 

The second cluster lies close to the first and mainly deals with the behavior of users and streamers. The link with the first cluster is established through the different motivations that give rise to widely differing behavior [85]. The topic is extremely complex as it relates to behaviors, for example, the intention to purchase and the sending of virtual gifts [100], the identification of paying viewers [107], subscription [68] and impulse buying [66], or wishful identification and emotional engagement as a predictor of many of these behaviors [70]. It also tackles viewers’ information activities [18], or negative behavior such as online harassment [106], excessive use of live streaming services [67], aggressive attitudes in discourse or speech [116], and even the negative effects of streaming on streamer performance [99]. A distinctive subject within this cluster refers to the recommendation systems for choosing content [73] based on the users’ behavior, preferences, or degree of engagement [96,143]. 

In the third cluster, which is aligned with the first two, the topics of the domain extend to the monetization of the activities of streamers and platforms [82,118]. It deals with topics such as user loyalty [63], covering the need for streamers to build and maintain audiences [102,128,129], activities that foster engagement [33], or systems to increase the number of viewers [129]. The cluster contains papers that develop tools to offer additional information to the user, thereby improving their experience and enhancing the connection between streamers and watchers [128]. We also found the most important theme of the sample analyzed, represented by the keyword Twitch, which was the one that appeared most frequently. This core term acts as a nexus linking the six thematic clusters identified. The topics covered relating to this popular live streaming platform are very varied and fundamental to live streaming research. The term Twitch features particularly strongly in the subdomain or specialization focused on analyzing platforms. In relation to this node, comparisons with other streaming platforms [88] have been carried out, subcultures of players and viewers have been identified [89], and shared patterns of media consumption and production have been analyzed, [52] as well as the similarities and differences with fast-consumed repositories [90] or with other types of “static” content distribution platforms such as YouTube and Netflix [91]. In addition, Twitch has served as a reference to analyze traffic patterns [92], examine its business model and impact on the video game industry [8], explore its potential in terms of marketing [109] and education [71], and study its content policies [119].

**Figure 5:**
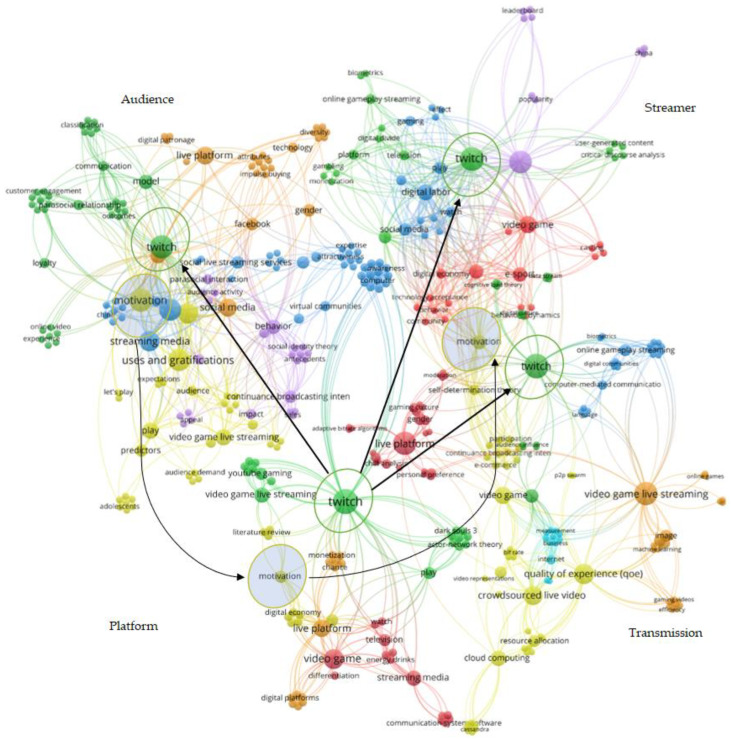
Connections between the different subdomains identified in the sample of documents.

The fourth cluster brings together topics related to the quality of experience (QoE) [77,94,141] and its assessment, video quality [20,135,140,145], or cloud computing [16,75]. As can be seen in Figure 4, this thematic cluster lies slightly farther away from the others. This could indicate a certain disconnect between research focused on motivations and behaviors, and those studies that attempt to tackle problematic issues relating to live streaming. However, identifiable thematic connections appear through the nodes of Twitch and motivation. Figure 5 shows the four subdomains represented individually and indicates the connections detected. This cluster is especially relevant in research focused on optimizing transmission, and it is here where the majority of technical documents can be found. There is a wide variety of topics addressed and a wealth of related applications being developed. Among the topics that have been covered, we found the development of functionalities to enhance communication [134,137], immersive user experiences through the implementation of multi-view systems [93], resource allocation to facilitate adaptive live streaming [141], and the application of the streamer’s biometric data to enhance the viewer’s experience [79]. Another significant group of papers in this cluster dealt with how to reduce bandwidth overheads and operating costs [16,131,133,138], broadcast times and latency [19], or metrics to optimize video quality [20].

The fifth cluster is linked to the social component of video game live streaming, media usage, and social media. The relationship with the first three clusters (C1, C2, C3) relates to the terminology used by Gros, Wanner, Hackenholt, Zawadzki, and Knautz [17] and Zimmer, Scheibe, and Stock [18], who view video game live streaming services as a social network (SLSSs). This group is characterized by the emphasis on the community elements of media enjoyment [86], thus offering a vision of these platforms as a new means of social communication [9]. It addresses specific topics such as the use and popularity of complementary social networks like YouTube or Instagram to gain followers [102], or the commoditization of the viewer’s social interaction while consuming media through gift-giving behaviors [112]. The general theme centers on the socio-motivational elements of uses and gratifications theory—that is, meeting new people, the search for interaction and social support, or feelings of belonging to a community [61]—or, as Lin, Bowman, Lin and Chen [62] point out, it deals with live streaming as a co-constructed social experience involving rich interactions between the content of the game, players, and viewers. Connections with the fourth and sixth cluster appear through studies focused on the popularity of live streaming channels [114] or spectator reactions [97]. 

The sixth cluster includes the most cross-cutting and current issues detected in all clusters, most notably the technology, the culture generated around live streaming communities, and more controversial matters related to gender, sexism, sexual content, and regulatory policies. In relation to the topic illustrated by the keyword “technology”, it can be seen that it is overly general; however, this node appears aligned with some of the documents on the professionalization of streaming content [10,54]. The topic is therefore more associated with the use of technological tools, mainly on the side of the streamer—that is, with the use of technological tools to facilitate streaming [10]—although it also extends to the use of fraudulent techniques such as chatbots to simulate an audience [78]. Furthermore, cultural aspects feature strongly in almost all of the research as these elements represent a substantial component of the studies on video games [52]. Such studies approach the topic from two perspectives. On one hand, there are descriptive studies, for example, Churchill and Xu [89] discussed subcultures of gamers represented in live streaming—casual, SpeedRunners, and competitive. On the other hand, some studies have delved into more contentious issues such as racism online [120], or discrimination on the basis of physical appearance or sex [123]. This cluster also includes all the topics related to gender issues, for example, the role played by gender when it comes to streaming [84] or doing e-sports [127]. It also shows connections with emerging issues such as content moderation [124], the processes giving rise to toxic technocultural discourse [125], or the analysis of user guides and the thin line separating freedom of expression from the use or abuse of inappropriate content [122]. 

## 4. Future Research Agenda

This article presents an exhaustive literature review carried out using the PRISMA protocol. The combination with BPNA and the science mapping of the most significant research topics represents the first attempt at a comprehensive approach to video game live streaming research. After reviewing 111 documents, the results pointed to the communication process as the central axis around which the study of this new mass phenomenon revolves. It was thus possible to adopt the elements of the model proposed by Shannon [21] to identify the structure of this nascent research domain [45,46]. The findings revealed four specializations or subdomains: studies focused on the transmitter or streamer; the receiver or the audience; the channel or platform; and the transmission process. These four specializations add to the accumulated knowledge through the development of six core themes that emerge: motivations, behaviors, monetization of activities, quality of experience, use of social networks and media, and gender issues. In line with the findings of the meta-review, we can see the emergence of interesting new directions and ample opportunities for future research, which are discussed below.

Relating to the audience, three basic dimensions have been studied: information, entertainment, and socializing [17]; these refer to the benefits perceived by the viewer, according to theories grounded in behavioral studies such as the uses and gratifications theory [7,11,17,18,59,60,61,62,63,64]. Among the most significant findings is the fact that the search for information is positively associated with the number of hours spent viewing video games [59]; indeed, it is one of the strongest predictors of the use of live streaming platforms [64]. It has also been found that social integration predicts subscription behavior [59] and that the viewer’s identification with streamers and audience groups is positively linked to the intention to continue watching live streams [65]. Furthermore, studies have uncovered connections between the genre of video games played, the content streamed, and the viewer’s gratification [60]; motivations and money spent [104]; and the size of the channel and viewers’ social engagement [61]. Another notable aspect are the links between social issues and the use of streaming platforms [86]. However, although viewer motivation was one of the most extensively-analyzed topics in the sample, most of the related studies revealed certain contradictions among the findings, which point to the need for further study and to consider other variables that may influence viewer motivation [11,27]. A clear example is the possible differences in motivation according to the gender of users or streamers. In this regard, Gros, Hackenholt, Zawadzki, and Wanner [104] found that gender did not seem to play an important role in the motivations for use and money spent. Similarly, other studies found no moderating effects of sex on the motivations for the use of streaming platforms [64]. However, there have been some studies that attest to clear differences depending on whether a viewer is watching a source from the same gender or different gender in terms of credibility [105], comments made and received in the field of e-sports and beyond [33,127], and even in the way of sending or receiving messages [84]. Therefore, there is a need to delve deeper into the debate on sources of motivation, with more in-depth theoretical and empirical exploration. The related models developed have to be applied in different cultural contexts, and new variables widely used in the field of video game studies need to be incorporated [150].

Regarding the streamer, we found studies exploring the characteristics, behaviors, and challenges of live programming, revealing that streamers are motivated by the desire to share knowledge, socialize, and build identities online, taking on challenges related to the tools available and maintaining viewer engagement [85]. In this vein, Zhao, Chen, Cheng, and Wang [53] and Torhonen, Sjoblom, Hassan, and Hamari [54] examined the motivations for creating content, distinguishing between intrinsic motivations—enjoyment and socializing—and extrinsic motivations—associated with work activities such as earning an income or gaining prestige—although it seems that the latter are still less important drivers of content creation. Aligned with this theme, we observed the emergence of a core of significant studies that approached live streaming as a new form of paid work [10,58]. They explored the way in which the activity was carried out and the tools available for professionalizing this hobby [10], affective and immaterial labor on these platforms including being friendly, soliciting donations, building a community and engaging audiences [83], and even the economic and inclusion opportunities for people with disabilities and other health issues [57]. This line of research seems especially interesting; indeed Guarriello [56] calls for studies to focus on people who are willing to give up secure, traditional jobs in order to turn their hobby into a profession without any kind of social safety net, and ultimately commit to continuous streaming. In this regard, it has been observed throughout the review that there are hardly any studies from the field of management or business, and very rarely has any research focused on this labor activity as a way of starting a business and entering the labor market [55]. Accordingly, there is a need to gain a better understanding of this issue with new approaches from these areas, to incorporate approaches such as the careers perspective [151], and to analyze transitions from other activities to streaming and vice versa. In turn, in the research focusing on the streamer, we found quantitative empirical evidence and attempts to explore certain issues on the horizon such as how these new professionals manage success and failure [58]. 

Relating to the research on platforms, it was shown that live streaming has become a novel hybrid of conventional television, YouTube, and a social network, whose main (though not entire) content is video games. As Chen and Xiong [117] revealed, we are witnessing dizzying changes in the different business models, most significantly regarding the transitions to other types of content. New live channels for music, sports, travel, gaming, education, and even academic research are continually emerging [115]. From this perspective, managing the diversification of activities [152] is one of the most important challenges for the future. Although the major impact of the current pandemic on live streaming has yet to be fully gauged, professionals from other fields have already capitalized on the channels provided by these platforms to release music albums or perform world premieres; examples include the Code Orange album release on Twitch [153] or the live-streamed Travis Scott concert in Fortnite. It remains to be seen how copyright [144] or sponsorship [68] will be managed from now on. The opening up of content, and the professionalization of activities with the arrival of influencers from other fields represents a challenge for the platforms’ strategic communication [154]. We have yet to see how potential cross-platform “brain drain” will be managed in the future, or how content policies will be adapted [119]. Another aspect of particular interest that has not been widely analyzed thus far is the evolution of the relationship between the video game industry and the live streaming industry [155]. 

Finally, as far as streaming itself is concerned, the possibilities are extraordinary. In the near future, a great deal of attention will be paid to the subscription models for cloud gaming platforms such as Stadia, Xcloud, or Amazon Luna, which offer catalogues of video games and incorporate functionalities that further facilitate live streaming. The management of communication and the broadcast of messages in group chats [111] pose new challenges: how to manage messages [121], the prediction of popular events, the use of emojis [97], or the information overload that will increasingly affect online group communication [113]. There are also certain unknowns regarding whether it will be possible to incorporate virtual reality into live streaming.

## 5. Conclusions

This review underlines the complex, multidisciplinary nature of the phenomenon of video game live streaming. The differences in citation patterns, methodologies, and theoretical frameworks used may stem from the different areas of research involved in each of the identified specializations, although they denote common characteristics of the research domain. Strong links were observed between subdomains centered on the streamer and the viewer; however, they appear to be somewhat disconnected from the specializations focused on the platforms and transmission processes. This disconnect will no doubt be resolved in the future as the volume of research increases and the related areas of knowledge are further democratized. The review of the documents in the sample provides a rigorous, detailed definition of live streaming services. This approach highlights the importance of gaining a greater theoretical understanding of the communication process and the interactivity entailed in this type of service. The review provides a significant reference for scholars interested in examining this new phenomenon. 

Although research on the live streaming of video games is awakening academic interest, the theoretical contributions developed are still limited, from its definition of the phenomenon and the elements involved, to its meaning in a new cultural and media context. The approach proposed here does not pretend to be unique or exclusive; it has attempted, through the compilation of accumulated knowledge, to show the basis for future research. Future works will need to incorporate new theoretical approaches from the field of videogame studies (e.g., the fit that the discussion between narrative or ludology has for research on live streaming [156], or the search for authenticity through the videogame [157]). On the other hand, the analyzed documents place the communication process as the central axis for video game live streaming, however, the present communication model including video game streaming expands Shannon’s perspective [21] used in our document classification. This insight, although limited, is a call for scholars in the field of communication, as this model has been criticized [158], expanded, and refined over the years, and as Al-Fedaghi [159] noted: *“Communication includes all aspects involved in the creation, export, import, and processing of artifacts used to link objects in the world. The study of communication encompasses all features of a communication system, including its technical, personal, social, and organizational forms”*, very much present elements in the live transmission of video games. 

However, the results presented here are not free from limitations, which relate to the studies included in this review. The first limitation stems from the use of a single data source to obtain the final sample, which means relevant documents not indexed in the WoS were excluded. Although this was a self-imposed limitation to avoid mixing citation formats or subject categories, it opens the door to new analyses with other databases that can help test the findings obtained here. Second, although a rigorous procedure was employed in order to avoid possible biases, the coding of documents and their classification inevitably entails a certain degree of subjectivity that could distort some of the assessments. However, we believe that the combination of methods helps to minimize the impact. Third, this study presents a snapshot of the situation; the evolution of the research domain thus remains to be studied, calling for future reviews that can capture the dynamism of all the issues presented here.

We conclude that there is still a great deal of heterogeneity in the results obtained in the different thematic clusters and specializations identified. Given the increasing academic interest in live streaming services, there is a need to create a common basis for research that can guide theory and practice and enable interdisciplinary collaboration to support the future growth of this emerging research domain.

## Figures and Tables

**Table 1:** VOSviewer configuration.

Item	Characteristic/Value
Type of analysis	Co-occurrence
Unit	All Keywords
Counting method	Full counting
Normalization Method	Association Strength
Layout	Attraction = 2/Repulsion = 0
Clustering	Resolution = 1.00/Min. Cluster size = 10
Visualization Scale	network and overlay = 1.27
Weights	Occurrences
Labels size variation	Min. Strength = 0/Max. Lines = 500
Minimum number of occurrences of a keyword	Main sample (n = 111) = 2
	Streamer sample (n = 19) = 1
	Audience sample (n = 25) = 1
	Platform sample (n = 17) = 1
	Transmission sample (m = 50) = 1

**Table 5:** Thematic clusters identified in the research domain on video game live streaming.

Cluster/Color/Label	Nº Keywords	Keywords (Links, Total Link Strength, Occurrences)
C1/red/Motivation	18	Video game (56, 146, 25); Motivation (47,110,17); E-sport (35,61,10); Play (33, 68, 10); Digital labor (19, 39, 5); Television (21, 36, 5); Watch (23, 40, 5); Digital Economy (16, 23, 3); Engagement (20, 26, 3); Predictors (13, 17,3); User-generated content (14, 16, 3); Communication (9, 10, 2); Game Studies (10, 12, 2); New media (18, 23, 2); Parasocial interaction (11, 11, 2); Parasocial relationship (10, 11, 2); Social presence (9, 9, 2); Work (8, 9, 2)
C2/green/Behavior	15	Video game live streaming (11, 66, 18); Youtube gaming (30, 49 7); Behavior (26, 32, 5); Continuance broadcasting intention (14, 18, 4); Live broadcast (3, 3, 3); Participation (22, 24, 3); Self-determination theory (21, 24, 3), Audience (11, 12, 2); Online games (4, 5, 2); Personal preference (3, 4, 2); Recommendation system (3, 4, 2); User acceptance (14, 15, 2); Virtual communities (11, 11, 2)
C3/blue/Monetization	13	Twitch (65, 173, 40); Online gameplay streaming (15, 20, 5); Monetization (21, 21, 4); platform (18, 23, 4); Gaming (16, 16, 3); Loyalty (19, 19, 3); Biometrics (6, 6, 2); Computer-mediated communication (4, 4, 2); Gambling (9, 12, 2); Gamification (16, 16, 2); Politics (8, 10, 2); Viewer engagement (9, 10, 2); Wearable technology (3, 6, 2)
C4/yellow/QoE	13	Quality of experience (14, 23, 8); Crowdsourced live video (13, 24, 7); Cloud computing (7, 13, 5); Model (24, 33, 5); Image (4, 7, 3); Impact (18, 18, 3); Quality assessment (4, 9, 3); Resource allocation (4, 8, 3); Video quality (4, 7, 3); Cognitive load theory (6, 7, 2); Experience (15, 15, 2); System (10, 10, 2)
C5/purple/Media usage	13	Streaming media (45, 109, 19), Media usage (36, 90, 9); Social media (38, 79, 9); Uses and gratifications (35, 71, 9); User behavior (21, 28, 4); Facebook (20, 26, 3); Internet (16, 19, 3); Social live streaming services (11, 18, 3); China (11, 11, 2); Information behavior (13, 14, 2); Intention (16, 23, 2), measurement (8, 8, 2); Popularity (9, 9, 2)
C6/Orange/Gender and Contents	13	Live platform (56, 11, 30), Gender (26, 42, 7); Technology (27, 24, 5); Gaming Culture (9, 14. 3); Platform regulations (8, 14, 3); Sexual content (8, 15, 3); Chat analysis (4, 5, 2); Diversity (5, 5, 2); Online community (3, 5, 2); Sexism (9, 11, 2); Video popularity prediction (2, 3, 2); Viewer participation (5, 6, 2); Women (5, 9, 2)

## Data Availability

The data used to support the findings of this study are available from the corresponding author upon request.

## Appendix A

**Table A1:** Top ranking documents in each of the specializations or subdomains identified.

Item	Focus *	Method *	Theoretical Framework	Goals	Data	Rk. Citations	Rk. Count 2013	Rank Count 180 d
[53]	S	Qn structural equation modeling	self-determination theory	Determine the factors influencing live streamers’ intentions to continue broadcasting on Twitch.	n = 306 Taiwanese live streamers on Twitch	6	5	5
[101]	S/A	Qn case study	No definition provided. Previous Research	Analyze how the streamers and spectators behave and further characterize the live-streaming community.	a set of 54, 246, 484 tuples in the format < user, channel, date, time, action, chat >	10	87	23
[10]	S/A	Ql ethnographic	affordance theory	Identify what practices and elements individual Twitch streamers utilize and what affordances these practices create for the streamers and viewers.	100 different streamers on Twitch	16	19	24
[7]	A	Qn structural equation modeling	uses and gratifications theory	Examine why people watch eSports on the Internet.	people who have watched eSports online (n = 888)	1	1	1
[59]	A	Qn structural equation modeling	uses and gratifications theory	Study the general gratifications that people derive from watching online streaming content and the differences in various streaming content.	n = 1091 users	2	15	3
[61]	A	Qn Multiple and ordinal linear regression analyses	uses and gratifications theory	Explore social motivations and types of Twitch live-stream engagement and how the relationship between live-stream viewer motivations and indicators of engagement vary by channel sizes.	n = 2227 Twitch users	3	2	4
[52]	P/A/S	M Descriptive Statistics and Nvivo	multidisciplinary framework	Examine the relationships among production, consumption and practice on Twitch.tv	n = 16 live streaming videos/ n = 96 Twitch users	9	30	6
[71]	P/A/S	Qn laboratory experimental design/analysis of covariance	Cognitive load theory	Examine the efficacy of Twitch as a learning platform.	n = 350 participants	14	42	17
[91]	P/A	Tc Python crawler/Descriptive statistics/graph construction techniques	No definition provided. Previous Research	Explore the Twitch infrastructure to understand how it manages live streaming delivery to an Internet-wide audience.	API Twitch	17	49	52
[112]	T/A	Qn logistic and negative binomial regressions	No definition provided. Previous Research	Study the effect of viewer engagement on gifting items to a streamer in a live video streaming.	2,294,837 viewers over a three-month period	7	8	9
[16]	T	Tc Operational Cost Model/Delay Model/QoE Model/cost-effective online algorithm	Lyapunov optimization theory	Examine the problem of cost-effective adaptive live game video streaming from the perspective of CLGVS service providers.	Twitch Dataset to simulate the behaviors of gamers and viewers	12	34	31
[121]	T	Ql Multi-column transcription scheme, which includes the broadcaster’s spoken language & embodied conduct, the audience’s written chat messages as well as an annotation of game events	No definition provided. Previous Research	Determine what a transcript should look like in order to systematically account for the activity and the cross-modal communication between broadcaster and audience. Determine how the unfolding of the activity influences the cross-modal discourse during online live streaming.	n = 6 streamers 12 h of recording (Three popular games: League of Legends, FIFA and World of Warcraft)	13	63	22

* Focus = Transmitter/Streamer (S), Receiver/Audience (A), Platform (P), Transmission (T); Method **=** quantitative (Qn), qualitative (Ql), mixed (M), technical studies (Tc).

**Table A2:** Journal of publication, journal quartile and citation index in Web of Science (WoS) core collection.

Doc	Journal/Conference/Book	JCR Category */Quartile or Book/Conference	WoS Core Collection **
[10,59,60,61,65,69,70,71]	Computers in Human Behavior	Psychology, Experimental (Q1)/Psychology, Multidisciplinary (Q1)	SSCI
[93,117,145]	IEEE Access	Computer Science, Information (Q1)/ Engineering, Electrical & Electronic (Q1)/Telecommunications (Q2)	SCIE
[16,114,148]	IEEE Transactions on Circuits and Systems for Video Technology	Engineering, electrical & electronic (Q1)	SCIE
[9,57,58]	Information Communication & Society	Communication (Q1)/Sociology (Q1)	SSCI
[80,96,149]	Multimedia Tools and Applications	Computer Science, Information Systems (Q3)/Computer Science, software engineering (Q2)/Computer Science, Theory & methods (Q2)/Engineering, electrical & electronic (Q2)	SCIE
[56,119,126]	New Media & Society	Communication (Q1)	SSCI
[17,18,91,104,111,133,136,142]	Lecture notes in computer science (Proceedings papers)	Computer Science, Theory & Methods (Q4)	CPCIS/CPCISS&H
[90,95]	ACM Transactions on Multimedia Computing Communication and Applications	Computer Science, Information Systems (Q2)/Computer Science, Software Engineering (Q1)/Computer Science, Theory & Methods (Q1)	SCIE
[98]	Computer Networks	Computer Science, Hardware & Architecture (Q2)/Computer Science, Information Systems (Q2)/Engineering, Electrical & Electronic (Q2)/Telecommunications (Q2)	SCIE
[55,84]	Convergence the international Journal of Research into New Media	Communication (Q2)	SSCI
[123]	Critical Studies in Media Communication	Communication (Q2)	SSCI
[125]	Discourse Studies in the Cultural Politics of Education	Education & Educational Research (Q2)	SSCI
[62]	Entertainment Computing	Computer Science, Cybernetics (Q4/Computer Science, interdisciplinary (Q4)/Computer Science, software engineering (Q3)	SSCI/SCIE
[86,99]	Games and Culture	Communication (Q3)/Cultural Studies (Q1)	SSCI/A&HCI
[19,138]	IEEE Transactions on Multimedia	Computer Science, Information systems (Q1)/Computer Science, software engineering (Q1)/Telecommunications (Q1)	SCIE
[76]	IEEE-ACM Transactions on Networking	Computer Science, Hardware & Architecture (Q1)/Computer Science, theory & methods (Q1)/Engineering, Electrical & Electronic (Q2)/Telecommunications (Q2)	SCIE
[72]	Information Visualization	Computer Science, software engineering (Q3)	SSCI/SCIE
[118]	International Gambling Studies	Substance Abuse (Q2)	SSCI
[11,64]	International Journal of Environmental Research and Public Health	Environmental Sciences (Q2)/Public, Environmental & Occupational Health (Q1)/Public, Environmental & Occupational Health (Q2)	SSCI/SCIE
[20]	International Journal of Network Management	Computer Science, Information Systems (Q4)/Telecommunications (Q4)	SCIE
[7,54]	Internet Research	Business (Q1)/Computer Science, Information Systems (Q1)/Telecommunications (Q1)	SSCI/SCIE
[67]	Journal of Behavioral Addictions	Psychiatry (Q1)	SSCI/SCIE
[75]	Journal of Cloud Computing Advances Systems and Applications	Computer Science, information systems (Q2)	SCIE
[141]	Journal of Network and Computer Applications	Computer Science, Hardware & Architecture (Q1)/Computer Science, Interdisciplinary Applications (Q1)/Computer Science, Software Engineering (Q1)	SCIE
[121]	Journal of Pragmatics	Linguistics (Q2)	SSCI/A&HCI
[105]	Journal of Research in Interactive Marketing	Business (Q2)	SSCI
[127]	Journal of Sport & Social Issues	Hospitality, Leisure, Sport & Tourism (Q3)/Sociology (Q2)	SSCI
[8]	Media Culture & Society	Communication (Q2)/Sociology (Q2)	SSCI
[81]	Multilingua-Journal of Cross-Cultural and Interlanguage Communication	Linguistics (Q3)	SSCI/A&HCI
[139]	Peer-to-Peer Networking and Applications	Computer Science Information Systems (Q2)/Telecommunications (Q2)	SCIE
[33]	PLOS One	Multidisciplinary Sciences (Q2)	SSCI/SCIE
[109]	Public Health Nutrition	Nutrition & Dietetics (Q2)/Public Environmental & Occupational Health (Q2)	SSCI/SCIE
[113]	Royal Society Open Science	Multidisciplinary Sciences (Q2)	SSCI/SCIE
[115]	Science	Multidisciplinary Sciences (Q2)	SSCI/SCIE
[82]	Social Media + Society	Communication (Q1)	SSCI
[66]	Sustainability	Environmental Sciences (Q2)/Environmental Studies (Q2)/Green & Sustainable Science & Technology (Q3)/Green & Sustainable Science & Technology (Q3)	SSCI/SCIE
[53,112]	Telematics and Informatics	Information Science & Library Science (Q1)	SSCI
[83]	Television & New Media	Communication (Q4)	SSCI/A&HCI
[63]	Cyberpsychology Behavior and Social Networking	Psychology, social (Q2)	SSCI
[52]	Journal of Gaming and Virtual Worlds	-	ESCI
[120]	Book: Digital Sociologies	Book chapter	BCISS&H
[6,23,68,73,74,77,78,79,85,87,88,89,92,94,97,100,101,102,103,106,107,108,110,116,122,124,128,129,130,131,132,134,135,137,140,143,144,146,147]	Conferences	Proceedings papers	CPCIS/CPCISS&H

* JCR = Journal Citation Report; ** SSCI:Social Sciences Citation Index; SCIE: Science Citation Index Expanded; A&HCI: Arts & Humanities Citation Index; BSCISS&H: Book Citation Index Social Sciences & Humanities; CPCIS: Conference Proceedings Citation Index Science; CPCISS&H: Conference Proceedings Citation Index Social Science & Humanities.
